# ‘Life has become a lot easier’—the acceptability of the treatable traits clinic experience to people with bronchiectasis

**DOI:** 10.1080/07853890.2026.2741976

**Published:** 2026-10-06

**Authors:** Kirsty E. Krieg, Annemarie L. Lee, Marita J. Dale, Tiffany J. Dwyer, Zoe J. McKeough

**Affiliations:** a Sydney School of Health Sciences, The University of Sydney, Sydney, Australia; b Department of Physiotherapy, Central Queensland Hospital and Health Service, Rockhampton, Australia; c Department of Physiotherapy, School of Primary and Allied Health Care, Monash University, Melbourne, Australia; d Department of Respiratory Medicine, Royal Prince Alfred Hospital, Sydney, Australia

**Keywords:** Comprehensive health care, multidisciplinary health team, patient experience, physiotherapy, quality of life

## Abstract

**Introduction:**

The treatable traits approach involves the identification of traits that are measurable and clinically relevant to the patient, which informs an individualized management plan. While improved quality of life and health outcomes have been demonstrated, the acceptability of this approach to people with bronchiectasis has not been explored. The aim of our study was to explore the acceptability of the treatable traits approach to people with bronchiectasis in a regional setting.

**Patients and Methods:**

An exploratory qualitative study. Interviews were conducted with adults who first attended an outpatient bronchiectasis clinic in regional Australia, which had implemented the treatable traits approach. The interview guide was developed to explore the seven components of the theoretical framework of acceptability. Engagement with the transcripts occurred using the six phases of thematic analysis to develop key themes.

**Results:**

Ten participants were included (age 37–82 years; female sex, 70%; Bronchiectasis Severity Index, mild, 20%; moderate, 50%; and severe, 30%). This study reports three themes. ‘A tool kit to support my health’ explores the contribution of individualized and multifaceted interventions to a perceived improvement in respiratory health. ‘Better health, better self’ captures the patient-reported impact of improved respiratory health on quality of life. ‘Some things don’t change’ describes the ongoing challenges for people living with bronchiectasis that are not impacted by the clinic interventions.

**Conclusion:**

People with bronchiectasis from a single regional Australian clinic described a highly valued and acceptable clinic experience, with perceived improvements in respiratory health and overall well-being despite significant ongoing impacts on daily life.

**Clinical trial registration:**

This study was registered in the Australian and New Zealand Clinical Trials Registry (https://www.anzctr.org.au/, ACTRN12620000377932)

## KEY MESSAGES

- When implemented by a physiotherapist and respiratory nursing team in a regional Australian setting, the treatable-traits clinical approach was acceptable and highly valued by people with bronchiectasis.
- Multifaceted, individualized treatment plans and collaborative partnership with clinicians optimized motivation to self-manage, improving perceived overall well-being beyond respiratory health
- This potential clinical model is acceptable to people with bronchiectasis in settings where specialist multidisciplinary clinics are not available.

## Introduction

Bronchiectasis is a syndrome [1], heterogeneous in clinical presentation and impact, characterized by symptoms of chronic cough, sputum production, and recurrent infection [2]. Disease heterogeneity presents a significant clinical challenge for successful treatment and improvement of the quality of life of people with bronchiectasis [3]. One approach to care is the treatable traits approach. Treatable traits are identifiable, measurable, clinically relevant and treatable features of disease, that when addressed have been shown to improve quality of life and health outcomes in other respiratory diseases [4]. This approach prioritises the patient’s goals and treatment preferences in the development of a collaborative management strategy [5]. Focused patient-centred care is core, with the clinician guiding care and decision making according to clinical guidelines and evidence-based practice.

The perceived acceptability of clinical interventions to the people receiving them is an important component of person-centred care [6] and successful intervention implementation [7]. Acceptability is defined as ‘a multifaceted construct that reflects the extent to which people delivering or receiving a healthcare intervention consider it to be appropriate, based on anticipated or experiential cognitive and emotional response to the intervention’ [7]. The theoretical framework of acceptability (TFA) proposes seven components to acceptability: affective attitude, burden, ethicality, intervention coherence, opportunity costs, perceived effectiveness and self-efficacy [7]. There is significant overlap between concepts of patient satisfaction and experience with the acceptability of interventions. The component ‘affective attitude’ relates to ‘how an individual feels about the intervention’ which could include a person’s satisfaction/dissatisfaction with an intervention, but is distinct from satisfaction in that it can be measured prospectively when designing interventions [7]. A person’s experience of a healthcare intervention relates to four components of the TFA (affective attitude, burden, opportunity costs and perceived effectiveness), but is not a distinct separate construct [7].

Care approaches that are acceptable to patients facilitate motivation to engage in self-management strategies [8]. For people with bronchiectasis, it is not only symptoms and recurrent infection but also a sense (or loss) of control that greatly impacts quality of life and well-being [9]. Personalized approaches are preferred by people with bronchiectasis and healthcare providers [8], particularly with regard to airway clearance therapy [10] and disease-specific education [11].

While the delivery of evidence-based care is a priority for all communities [12], access to guideline-informed care, such as disease-specific education [13], specialized services [14] and peer support [13] can be limited or challenging in rural and regional settings. The implementation of a treatable traits approach to care within a bronchiectasis clinic in regional Australia with a physiotherapist and nursing team led to a significant improvement in quality of life and a reduction in exacerbation frequency in people with bronchiectasis [15]. This was achieved with a low-resource, pragmatic implementation of the treatable traits model, with the potential for application in other similar regional or rural settings.

The aim of our study was to explore the acceptability and experience of a regional outpatient bronchiectasis clinic using a treatable traits approach to personalize care in people with bronchiectasis.

## Methodology and methods

This was an explorative qualitative study conducted according to the Standards for Reporting Qualitative Research (SRQR) [16]. The epistemological view taken in this study was a form of social constructionism, where we considered the acceptability of the clinical experience to be constructed through the engagement of people with bronchiectasis with the clinical model [17]. We considered the acceptability of the clinic relative to the social context in which it was delivered [17]. In this study, the clinical context was regional Australia, greater than 700 km from a tertiary specialist center, with local access to respiratory medical care from either a private respiratory, a general public physician, or a general practitioner. Participants had not received specialist care in the previous 12 months, and the clinic was led by a physiotherapist and nursing team using a pragmatic, low-resource approach (Supplementary File S1). Participants were referred to other services/health professionals depending on the traits identified (e.g. pulmonary rehabilitation). Further detail regarding the clinic implementation is outlined in [15]. We believe that participants each made sense of the acceptability of the clinic in different ways relative to past experiences, rather than there being a singular ‘reality’ [17]. We considered that the values and experiences of the researchers as physiotherapists influenced both the design of the interview questions and reading of the transcripts. Four members of the research team had significant clinical and research expertise, primarily in a metropolitan setting in bronchiectasis, chronic obstructive pulmonary disease, and cystic fibrosis. One researcher has lived and worked in regional Australia for over 15 years, placing importance on access to evidence-based care for people living outside urban areas, with clinical expertise in acute and community settings. This researcher was also a physiotherapist who implemented the treatable-traits approach in the bronchiectasis clinic. The perception of acceptability described by individuals with bronchiectasis was interpreted using this lens.

### Ethical considerations and clinical trial registration

Ethical approval for this study was received from the Central Queensland Hospital and Health Service (CQHHS) Human Research Ethics Committee (HREC/2020/QCQ/61455). All participants provided written informed consent to participate in the study and to publish de identified study results.

This study was registered in the Australian and New Zealand Clinical Trials Registry (https://www.anzctr.org.au/, ACTRN12620000377932).

### Recruitment and participants

Adults with bronchiectasis who were attending the outpatient bronchiectasis clinic in Rockhampton, Australia, for the first time between January 2021 and March 2023 were invited to participate in the study. Information on study participation was distributed when people were referred to the clinic. Recruitment was consecutive and occurred before the first clinic appointment. Individuals were excluded if they had attended a specialist bronchiectasis clinic with a respiratory physician or respiratory nurse practitioner and a physiotherapist 12 months prior to study recruitment. Demographic information was collected at the time of obtaining the participant’s consent. Information was collected on age, sex, dyspnea [18], Bronchiectasis Severity Index (BSI) [19], exacerbation frequency, annual hospitalization (days/year), race, work status, highest education level, and other chronic conditions experienced by the participants (Table 1).

To determine the sample size of our study, we employed the concept of ‘information power’ [20]. We considered the aim of our study and the participant group to be narrow and specific, with a focus on adults with bronchiectasis who attended a single clinic in regional Australia for the first time. The interviewer had over 10 years of experience in interviewing and counselling people with chronic diseases to improve their quality of life and well-being. In choosing thematic analysis, we considered that our study would explore different views of acceptability and clinic experience and detailed patterns of meaning that help understand the role of a treatable traits approach in improving health; however, we would not capture all possible experiences of such a model. We continued conducting interviews until two members of the research team determined that there was depth and richness in the perspectives and experiences of people with bronchiectasis living in a regional setting.

### The interviews

The interviews were face-to-face (*n* = 9) or *via* telephone (*n* = 1), conducted by MD, a social worker delivering outpatient services in chronic diseases, Rockhampton, Australia, and were up to 44 min in length. The interviewer was unknown to the participants and was not part of the clinic model, though worked in the same location as the clinical team. The interviews were scheduled after the second clinic appointment with the physiotherapist (i.e. the three-month review appointment), and they had completed at least one assessment with the respiratory nursing team. The interviews were conducted when the participant was well (i.e. did not experience a current exacerbation of bronchiectasis [21]. The questions were semi-structured and explored participant goals, the clinic experience, skills developed, and confidence in managing bronchiectasis. The questions were designed to explore the acceptability of the treatable traits approach according to the seven components of the theoretical framework of acceptability (TFA) [7]. The interview guide mapped to the TFA is included as Supplementary File S2.

### Data analysis

The interviews were audio-recorded and transcribed verbatim. The analysis of the interview transcripts followed the six phases of thematic analysis [22]. The process was reflexive and iterative, commencing with transcript familiarization and discussions between the two researchers. Repeated engagement and reflection occurred with the transcripts to capture the experiences of participants beyond semantics in the coding process. An ongoing discussion has enabled different perspectives to promote depth and breadth within the analysis through a process of inductive coding. Codes were clustered and re-clustered to develop a deeper understanding of the clinic experience and life with bronchiectasis of the participants. As clustering occurred, the initial themes and sub-themes were visually represented to ensure coherence across the narrative. The themes were developed and refined through re-engagement with the codes and transcripts to ensure that each theme captured a shared meaning centered around participants’ discussion of the clinic value relative to living with and managing bronchiectasis. The TFA informed the development of the interview guide, but consistent with thematic analysis [22] and an inductive approach, themes were not mapped back to the framework. At each stage of the analysis, discussion occurred with members of the research team to promote rigor in the interpretations of participants’ experiences. As the lead researcher, KEK also kept a reflexive journal to consciously reflect on her internal biases towards interpretation as a clinician implementing personalized bronchiectasis care in regional Australia. The second researcher involved in coding the interviews was not involved in the clinic design or implementation, to bring a neutral perspective to the analytic process and to minimize the impact of unconscious bias.

## Results

Ten people with bronchiectasis were interviewed (age 37–82 years; female sex 70%; BSI: mild 20%, moderate 50%, severe 30%; racial background: Caucasian 90%, other 10%; work status: 90% retired, 10% full-time; highest education level: 40% less than year 12, 40% Year 12/Associate Diploma/Vocational qualification, 20% bachelor degree/doctorate). Most participants (*n* = 9) lived with at least one comorbidity, with gastroesophageal reflux disease (*n* = 6) most commonly experienced, walked slower than their contemporaries due to dyspnea (*n* = 5), and experienced a median of one exacerbation in the 12 months prior to recruitment (Table 1 and Figure 1). The identified traits and treatments implemented in the study participants are summarized in Supplementary Table S1.

**Figure 1.**
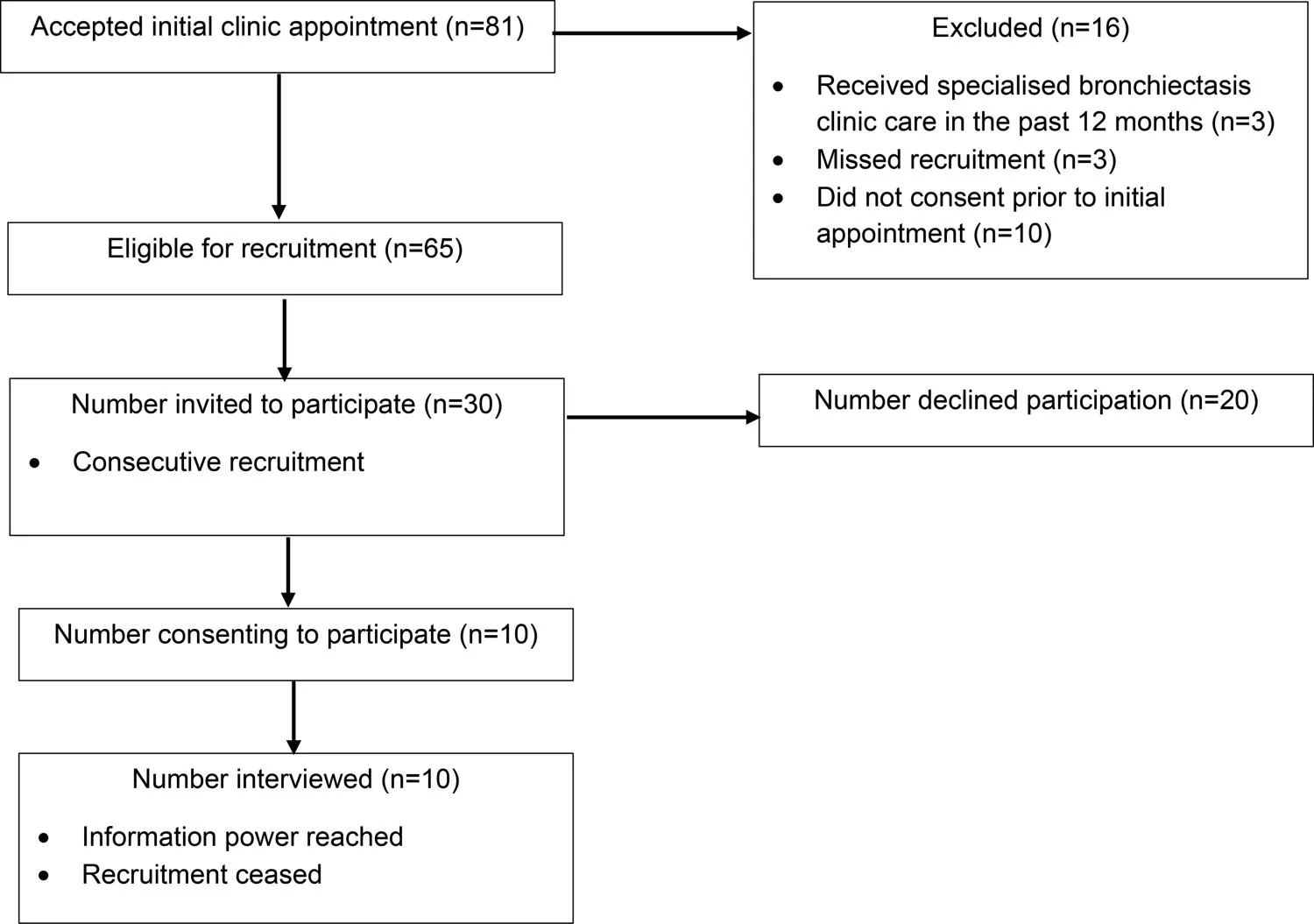
Participant flow for the semi-structured interviews.

**Table 1.** Characteristics of interview participants attending the bronchiectasis clinic, *n* = 10.

Characteristic	
Sex, n (%)	
Female	7 (70)
Age, *years,* mean (SD, range)	70 (12, 37–82)
Education level, n (%)	
Less than Year 12	4 (40)
Year 12/ Associate Diploma/Vocational qualification	4 (40)
Bachelor degree/Doctorate	2 (20)
mMRC Dyspnoea scale, n (%)	
0 Breathless with strenuous exercise	2 (20)
1 Breathless when hurrying on the level or walking up a slight hill	2 (20)
2 Walks slower than contemporaries on flat ground	5 (50)
3 Able to walk <100 yards or a few minutes on the level	0 (0)
4 Breathless with dressing or housebound due to breathlessness	1 (10)
Bronchiectasis Severity Index, n (%)	
Mild	2 (20)
Moderate	5 (50)
Severe	3 (30)
Comorbidities, median (IQR)	2 (1,2)
GORD, n (%)	6 (60)
COPD, n (%)	1 (10)
Sinusitis, n (%)	2 (20)
Heart disease, n (%)	2 (20)
Other chronic respiratory disease, n (%)	5 (50)
Cancer, n (%)	1 (10)
Other chronic condition, n (%)	2 (20)
No comorbidities, n (%)	1 (10)
Annual exacerbations in previous 12 months, median (IQR)	1 (0,1)
Annual days in hospital in previous 12 months, median (IQR)	0 (0,2)
Attended pulmonary rehabilitation in previous 12 months, n (%)*	0 (0)

COPD, chronic obstructive pulmonary disease; GORD, gastroesophageal reflux disease; IQR, interquartile range; mMRC, modified Medical Research Council; SD, standard deviation; * attendance of at least 12 supervised exercise sessions if referred to a pulmonary rehabilitation program in the 12 months prior to study enrolment

This study reports three themes (Table 2): 1) ‘A tool kit to support my health’ explores the contribution of different individualized clinical components to an outcome which is larger than the sum of its parts; 2) ‘Better health, better self’ captures the impact of improved respiratory health on overall wellbeing; 3) ‘Some things don’t change’ describes the many ongoing challenges when living with bronchiectasis that don’t change as a result of the clinic interventions. While the aim of the study was to explore the acceptability of the treatable traits clinic approach to people with bronchiectasis, the impact of bronchiectasis on everyday life was a dominant theme, and this perspective has been included to give context to the perceived benefits and experiences of the clinic. The theme-development process is outlined in Supplementary Figure S1. In the context of living >700kms from a specialist bronchiectasis clinic and not having received specialist care for bronchiectasis in the previous 12 months, negative views of the clinic were not expressed by any of the participants.

**Table 2.** Theme summary.

Theme	Characteristics
A tool kit to support my health	People with bronchiectasis describe the overall experience of the clinic as motivating and valuable to improving daily life. It is not only the strategies themselves, but the way they are individualised and combined that contributed to an improvement in symptom and health management.
Better health, better self	Improved mood, wellbeing and participation in life as a result of improvements in respiratory health.
Some things don’t change	The impact of bronchiectasis is significant in everyday life and this doesn’t necessarily change through improvements in overall health and quality of life.

### A tool kit to support my health

This theme centers on the idea that it is the way in which strategies are combined and individualized, which maximizes impact. Comprehensive, individualized care was described as building capability and enthusiasm for self-management. For some, the clinic was perceived as a valuable safety net against future health decline, with a desire to have ongoing access into the future.

People with bronchiectasis described how participating in the clinic interventions and developing relationships with staff at the clinic was a motivating and valued experience, facilitating a change in health and lifestyle behaviours which they had not previously prioritised.

I think (the clinic) just motivated me to pay more attention and to think more about my actual condition … improved my motivation to try and do something about it. (P08)

… Exercise I hate… just general lack of motivation to exercise … so I get enthused by the visits. And then I probably go home and do a bit of exercise where I don’t normally do it… It’s given me a bit more enthusiasm… I take every opportunity to try and get a better quality of life, particularly for my wife who has to put up with me… (P04)

I say to my kids… just think it’s part of my life, that mummy has to go and do her breathing. I know that this will keep me alive longer, so this is what I will do… with the benefit from the clinic (I can) manage the exercise program with clearing the lungs daily, just so… I can be as normal a lifestyle as possible. (P09)

People described feeling stronger together–attending the clinic and meeting other people with bronchiectasis provided a community that people could be part of, and this provided motivation to engage with the interventions provided at the clinic.

… I just never knew there was help for us, and I never knew so many other people had it. You always think you’re on your own. I knew I wasn’t the only one… it’s a really wonderful service, oh goodness me. (P05)

I need to get out of the house and to participate with others in doing things. I think… I’m used to being on my own. And it takes a courage to break out of that cycle… (P02)

There was great value placed on the different clinical components of the care delivered at the clinic. Establishing an effective airway clearance therapy plan was a priority as people experienced improvements in their health when a plan was implemented.

Well number one is the daily program to clear the lungs of mucus as much as I possibly can, I wouldn’t have had that without coming to the clinic… I would say at the moment I don’t even feel like I’m sick. I know I’m sick but I don’t- the airways are really so much clearer. (P09)

… we went away for just three days and in those three days I thought, oh don’t worry about the (airway clearance) exercises… But when I got home, I knew those three days were detrimental not having done the exercises, so I know the benefit of it… it’s just a bit of time out of your day, big deal. (P06)

… I have to use the nebulizer in the morning and night, and then I have to use an Aerobika. Between those sessions in the morning and nighttime, I definitely don’t cough anywhere near as much as I used to. (P10)

… they explained how to help yourself with your breathing. That’s the main thing; with your breathing you’ve got to know how to get the phlegm out of your chest… (P01)

Exercise was also perceived as an important piece of the puzzle, with people noticing that improvements in exercise capacity contributed to their overall well-being.

I think exercise might be the key. (P02)

(exercise) It helps with everything… it just made me, feel… um freer? … And I felt better. I feel like I can walk out better… Yeah, I think it’s helped me *(sic)* physical fitness. (P03)

There was a sense that bringing interventions together to form a bronchiectasis action plan assisted the self-management of bronchiectasis, by providing strategies to manage the disease that accommodated variability in symptoms and could limit disease progression, even though there is no cure.

I was a doctor and I’ve had this condition for 50 years. I had my first surgery when I was 20, so this is something I’ve lived with for a long time. It was stable until about five years ago and all of a sudden it’s gone pear-shaped. In some ways it’s a pity I didn’t meet up with (the) program four years ago because I may not have suffered as much perhaps damage as I have. (P08)

I think anyone who has this disease, that should be one of the first things (a bronchiectasis action plan), because it makes you feel a little bit like you’ve got control. If things go wrong you know what to look for, but you also know what to do. (P09)

… it’s about managing, it’s all about managing … I feel more confident, but I can’t, you can’t fix it … (P04)

Some described the clinic as a safety net and the importance of the relationship with clinical staff to feel confident in maintaining bronchiectasis management, while others described a feeling of being overwhelmed by their bronchiectasis when feeling unwell.

I don’t know when it comes (infection). I’ll probably panic. (P03)

I would like it (the clinic) just to keep going. I feel more confident. If it wasn’t, I don’t know like, when I have a problem, what do I do?… I just feel confident coming here and talking to (physio). If I’ve got a problem, I know my appointment’s coming up that I can ask (physio) about. (P10)

Across the various interventions implemented in the clinic, people with bronchiectasis emphasized that the individually tailored, comprehensive, and multifaceted nature of the interventions had a large impact on their management.

I just wanted to have a daily routine. I’m very young to have this disease and I work full-time and I’ve got two young kids, so I wanted a daily routine that was manageable and evidence-based, and realistically achievable because I know from my background that if you give people goals that aren’t achievable they just give up. I needed goals and a daily routine that I knew I could stick to… I think- by coming into the clinic I could actually be shown the techniques. I could practice them and have an expert guiding me in how I was doing things. Was it correct, and what could I do better? (P09)

I also have some other problems with ENT sort of things so (the clinic) actually referred me to a speech pathologist for that. So that’s been followed up on, and I’ve been referred on to an ENT to see if we can solve that issue, and that’s something (the clinic) picked up on… I think from the very first time I came I had everything explained so well to me, I can’t fault anything that was explained, I understood it very well. I asked questions that I got answers to that I wasn’t sure about myself but maybe the specialist himself hadn’t sort of fully covered for me. (P06)

### Better health, better self

The theme ‘better health, better self’ explored the concept that while people with bronchiectasis attended the clinic with different expectations of what they might gain from the experience, there were sometimes unanticipated benefits that went beyond improving respiratory health.

As improvements in respiratory health were realized, people with bronchiectasis described an improvement in mood and optimism for the future.

… my CT scan is really, really bad and I was fixating on that. My lungs are terrible, this is terrible, terrible, terrible, but (the clinic) helped me shift my mindset to, that’s just a photo but symptomatically you’re like a normal person. Forget about that and just work on what you can work on… (P09)

Life has become a lot easier really. (P01)

There was also a shift in perspective and engagement with life, a desire to participate, and be active, which had not been previously experienced.

In your lungs… you feel better and feel like doing more… well, before I was just sitting at home doing nothing and thinking that I was getting worse and now I feel that at least something is being done and I’m getting help and it makes me feel better in myself. (P01)

I feel more healthy… I have a lot more stamina. I think in general, I feel more positive. And whether that’s a reflection of the fact that I am physically better… I do know that when I’m mildly unwell, and there have been times where I’ve been mildly unwell for relatively long periods of time, it really affects my mood. (P08)

People with bronchiectasis described improved control in their own lives, which contributed to an experience of improved well-being and positive self-thought.

… before I went to the clinic I was just researching, trying to get things in my head of how I could get control, because I felt very out of control… A year ago I felt like my life was spiralling but I don’t feel that way anymore. (P09)

… I was getting to that stage too, if I had to go out for lunch with friends, it’s sort of in my mind, I hope I don’t have that terrible phlegmy cough. Please don’t let that happen. I don’t have that fear now, because once I clear it in the morning and at nighttime, it’s pretty good… People have noticed that I don’t have that cough, which I’ve had for many years… I’m really happy with what I’m doing now. (P10)

For others, a contradiction was described regarding the improvement in symptoms after attending the clinic. It was perceived that a reduction in symptoms might also indicate something harmful or unknown, as the symptoms are, in some ways a comfort.

I am caught between the devil and the deep blue sea, I’ve got to get that mucus from the outer reaches of the lungs into the middle… the more you get out, the less bad germs are in your lungs. So, in order to do that, like a good day is when you don’t hack (cough), but that’s really a bad day, because you haven’t got rid of the bad bugs… So I’ve trained me *(sic)* mind to accept that it’s a good day when you have a bad day… (P04)

### Some things don’t change

Some aspects of everyday life do not change despite having a toolkit, individualized support, and improvements in overall well-being.

People describe a life that is more constrained with bronchiectasis, that a sense of self is lost as they cannot be the person they would like to be.

It’s a very limited life mine… I kidded myself that I could still manage it, and I did do a holiday and it was a disaster… I can’t even go like local with other friends because you can’t share accommodation with anybody because if you cough, it’s just, and I’ve just realised that now I’ve got to, Ive given that up. (P03)

… I’m pissed off that I can’t travel. I got lots of money. But I can’t travel… I can only do about a quarter of what I could do in the shed and other things and like I used to trim the hedges. I can’t do that anymore. I stop three times to take the bin out. And that’s just life… the only time I go out is to go to the fucking doctor’s. Truly. (P04)

… if I go to the movies, I start coughing. And there’s nothing worse than a cougher in a movie is there? (P02)

For some, there was a strong sense of a life that was wasted or without usefulness.

… I have very rarely pushed myself to unthinkably extreme levels, and maybe I’ve missed out on a bit of life. (P08)

… If I was a horse they would shoot me I’m sure. (P02)

A key challenge described by people with bronchiectasis is that every day can look different. It is difficult to predict what is ahead, which can have a significant impact on daily life.

Having those lung infections kind of sets you back socially, emotionally, physically… Well, and here I say I’m well. I might have one good day and then you might have another bad day. I just feel I can’t do a lot, and then the next day you get up and start again. So, it’s up and down, it’s variable… (P05)

## Discussion

This is the first study to explore the acceptability and experience of attending an outpatient bronchiectasis clinic, implementing a treatable traits approach to care, for people with bronchiectasis in a regional setting. It is important to understand the ways in which this clinic model is perceived and experienced by people with bronchiectasis, particularly regarding the appropriateness of clinical care to improve daily life. Multifaceted and individualized care has been described as a crucial part of the puzzle to improve health. The experience in this regional setting was overwhelmingly positive, with people describing a larger impact on health than anticipated before attending the clinic, both from a respiratory health and overall well-being perspective. Despite this experience, there was still a part of life that did not change for some, with the sense that life could have been more fulfilling without the impact of living with bronchiectasis. While there are days or times when life is much easier, there are also times when bronchiectasis is a significant burden.

Key components of programs that improve adherence include the coordination of care to enable a person-centered approach and a focus on self-management and education [23]. In our study, people with bronchiectasis described the multifaceted interventions implemented by a physiotherapist and nursing team as being highly valuable and acceptable. Interventions such as airway clearance therapy, exercise, and bronchiectasis action plans were discussed as integral to having a sense of control over the disease. Treatment strategies, such as airway clearance, are not consistently performed in bronchiectasis, even in the presence of ongoing symptoms such as cough and sputum production [24, 25], hence seeing value in an intervention is important. If a treatment such as airway clearance therapy is perceived as necessary and important, there is a greater likelihood that the treatment will be performed as part of a self-management plan [26].

It was also the individualization of the plan components through a collaborative partnership with clinicians at the clinic, which was valuable and motivating to people with bronchiectasis. Optimizing motivation is key to improving people’s ability to self-manage bronchiectasis [8]. The importance of patient-clinician relationships in empowering people to self-manage has been described previously [27]. In people with chronic respiratory disease (including bronchiectasis), the relationship developed with local care providers has been described as facilitating the personalization of care, with this aspect of living in a rural community highly valued [13]. Regarding exercise, the language used to discuss exercise and the advice given are crucial to the perception of its value as an intervention [28]. It is also known that the individualization of education and care has a positive impact on the ability to self-manage bronchiectasis [29]. Of note, some people experienced this relationship as something to be continued and as a safety net for the future rather than feeling capable of managing alone, which has been reported previously [30]. This may reflect the varying nature of the disease over time, and the desire to have support for the unpredictable nature of bronchiectasis. While the benefits of a self-management program on quality of life and health care utilization in people with bronchiectasis have so far been inconclusive [31], positive outcomes may be possible with targeted, comprehensive strategies developed from a treatable traits approach that enables the personalization of care.

People with bronchiectasis also described unanticipated improvements in mood, engagement with life, and well-being that were not specifically targeted in this clinical model. Part of the improvement in well-being was described as attributable to improved control and respiratory health resulting from clinical interventions. There was also a contradiction described whereby the presence of symptoms was comforting, demonstrating the ability of the clinic education and clinician rapport to reframe lay illness perception, supporting clinic intervention coherence. Bronchiectasis is known to impact psychosocial well-being along with physical health [30, 8]. Higher rates of depression and anxiety have also been described in bronchiectasis and are associated with reduced quality of life [32, 33]. Notably, this was not associated with disease severity [33]. The diversity of disease experience, even in the presence of similar symptoms, is a feature of bronchiectasis [26, 34]. It is possible that a treatable traits clinic model, with a focus on patient-prioritized and individualized care, improves overall well-being by focusing on the diverse factors contributing to disease perception and symptoms. The individualized approach also supports the development of the clinician-patient relationship, which is foundational to building trust in interventions and confidence in self-management [30].

Bronchiectasis is disruptive to everyday life [30]. In this study, people with bronchiectasis describe significant ongoing impacts on daily life, even though attending the clinic contributed to having tools to support improvements in health. This is possibly a recognition of the complexity of factors contributing to health in an individual and the long-term nature of bronchiectasis.

### Strengths and limitations

Despite our attempts to provide access to guideline-informed care for all people with bronchiectasis in our community, there were no First Nations participants in this study, so their voices were not represented in this dataset. This may be because First Nations Australians are rarely referred to our clinic, which may reflect reduced awareness of the clinic as a referral pathway in some primary care settings. The clinic was designed with access to indigenous health workers and culturally designed educational resources, but people would not be aware of these resources prior to attending for the first time. We did, however, include people with bronchiectasis across a range of age, sex, disease severity, and education categories in a regional setting, where care access is known to be challenging [35]. Sufficient information power was confirmed through the initial coding of the interview transcripts, which demonstrated dominant themes regarding clinic experience and acceptability (e.g. airway clearance priorities and lifestyle impact). While further interviews would certainly deepen the understanding of treatable traits acceptability, it was determined by the research team that sufficient strength existed in the perspectives captured to describe clinic acceptability in a regional context. While information power was achieved in this context, it may not be fully reflective of acceptability of such a clinical model from all people with bronchiectasis.

We cannot exclude the possibility that participants’ knowledge that the study principal investigator would be involved in the analysis of the interview transcripts may have contributed to a social desirability effect, where participants were more likely to describe positive experiences of the clinic. However, there were participants who described not knowing how to self-manage the disease and significant ongoing life impacts, demonstrating that people were willing to describe challenges that did not improve following clinic attendance. People’s descriptions of their clinic experience reflect the perspective of those living with bronchiectasis who live at a significant distance (> 700 km) from specialist clinics and do not necessarily have the ability or desire to travel for healthcare. Our study also reflects the experience of a bronchiectasis clinic led by a physiotherapist and nursing team who took a pragmatic approach to implementing and personalizing care.

## Conclusions and practice implications

People with bronchiectasis attending an outpatient clinic in regional Australia implementing the treatable traits approach to care with a physiotherapist and respiratory nursing team described the experience as highly valuable and acceptable, supporting the delivery of care that met individual needs and improved overall health and wellbeing, despite ongoing long-term impacts from living with bronchiectasis. A treatable-traits approach delivered by a physiotherapist and nurses is one potential clinical model for the delivery of care, which was found to be acceptable to people with bronchiectasis in a single-setting based in a regional location where specialist multidisciplinary clinics are not available. Further research is required to describe the acceptability and experiences of First Nations Australians in a treatable traits clinical care approach, with the need for First Nations co-design to facilitate increased referral to bronchiectasis services and collaboration with First Nations researchers to lead research design and implementation.

## Supplementary Material

Supplementary Material_resubmission.docx

## Data Availability

The data supporting the findings of this study are available from the corresponding author upon reasonable request.
