# Supplementary material for: ‘Life has become a lot easier’—the acceptability of the treatable traits clinic experience to people with bronchiectasis
Source: Ann Med. 2026 Oct 6;58(1):2741976. doi: 10.1080/07853890.2026.2741976 (PMC13647353; doi:10.1080/07853890.2026.2741976)
Supplement: Supplementary file 1 [file IANN_A_2741976_SM5497.docx]

**SUPPLEMENTARY MATERIAL**

**Additional methods**

**File S1: A description of the treatable traits clinic approach implemented by a physiotherapist and nursing team in a regional bronchiectasis clinic**

**The initial physiotherapy clinic appointment (1 hour length, conducted by lead researcher)**

- Structured and standardised assessment of the traits present (e.g. mucus hypersecretion, reduced exercise capacity, dyspnoea).
- Collaborative discussion with the patient to determine which traits to prioritise for treatment and the possible treatments that could be implemented according to evidence-based guidelines in bronchiectasis (e.g airway clearance therapy, referral to pulmonary rehabilitation).
- Disease specific education, including exacerbation recognition and treatment goals
- Development of a bronchiectasis action plan, including the treatments prioritised with the patient.
- Correspondence to referring medical practitioner to outline the traits identified and bronchiectasis action plan developed.

**The initial nursing clinic appointment (up to 1 hour length, conducted by a respiratory nurse or nurse practitioner)**

- Structured and standardised assessment of the traits present (e.g. inadequate inhaler technique, low medication adherence, possible sleep apnoea).
- Collaborative discussion with the patient to determine which traits to prioritise for treatment and the possible treatments that could be implemented according to evidence-based guidelines in bronchiectasis (e.g inhaler review, timing of medications, self-management strategies).
- Disease specific education, including exacerbation recognition and treatment goals
- Development of a bronchiectasis action plan, focused on medication management strategies.
- Correspondence to referring medical practitioner to outline the traits identified, bronchiectasis action plan developed (inn collaboration with the strategies of the physiotherapist) and suggestions for medication review.

**Three month physiotherapy clinical review appointment (second appointment, 1 hour length, conducted by lead researcher)**

- Structured and standardised assessment of the traits present.
- Review of the traits prioritised and the treatments implemented.
- Practise and feedback regarding treatment technique/s (e.g. airway clearance therapy). Alterations made as required (e.g. change in airway clearance technique).
- Revision of the bronchiectasis action plan as required.
- Collaborative discussion of treatment goals and problem solving regarding challenges with self-management.
- Booking of future appointment, based on clinical need.

**File S2**: **Interview guide mapped to the theoretical framework of acceptability domains^*^**

^*^ The theoretical framework of acceptability domains and definitions are presented above each interview question in the blue text boxes (Sekhon, Cartwright and Francis 2017).

Intervention Coherence

The extent to which the participant understands the intervention and how it works

Ethicality

The extent to which the intervention has a good fit with an individual’s value system

1. A clinic is one way of providing strategies to help you manage your bronchiectasis. What were you hoping to achieve when you came to your first appointment?

Explore*:*

- *Who referred you?*
- *What did the referrer say about the reason to come to the clinic?*
- *Did you think coming to the clinic would be valuable to you?*
- *Was there anything you were not looking forward to?*
- *What goals did you set? Why these goals?*

Affective attitude

How an individual feels about the intervention

Ethicality

The extent to which the intervention has a good fit with an individual’s value system

2. What did you like most about coming to the clinic and why?

Explore:

- *Was it a good use of your time? In what ways? (think convenience, travel, costs, priority to you, impact on other activities) What parts were worthwhile?*
- *In what ways was it helpful? (helpful at the time or in the long-term?)*
- *What did you feel was the most important?*

Opportunity Costs

The extent to which benefits, profits or values must be given up to engage in the intervention

Burden

The perceived amount of effort that is required to participate in the intervention (e.g. time involved, cognitive load)

3. Is there anything about coming to the clinic that you dislike? If so, why do you dislike these things?

Explore:

- *What wasn’t helpful?*
- *What wasn’t a good use of your time?*
- *What sort of effort did coming require?*
- *What would you change if you could?*

Perceived effectiveness

The extent to which the intervention is perceived as likely to achieve its purpose (e.g. the likelihood a treatment will reduce infections)

Intervention Coherence

The extent to which the participant understands the intervention and how it works

Opportunity Costs

The extent to which benefits, profits or values must be given up to engage in the intervention

4. What do you think you gained from coming to the clinic?

Explore:

- *What did you learn that was helpful? (for example, ways of clearing sputum)*
- *What habits have you changed since starting at the clinic? (changes to exercise or activity, timing of doing your breathing exercises in the day, using a nebuliser)*
- *Did you adapt any of the advice to be specific to you?*
- *Which gains do you think will stay with you for the long-term?*
- *Did you have to give up anything to gain something?*

Self-efficacy

The participant’s confidence that they can perform the behaviour(s) required to participate in the intervention (e.g. confidence to use strategies for self-management)

Perceived effectiveness

The extent to which the intervention is perceived as likely to achieve its purpose (e.g. the likelihood a treatment will reduce infections)

5. Do you feel confident to manage your bronchiectasis with the help and advice you have been given? Would you recommend coming to the clinic to others with bronchiectasis? Why/why not?

Explore:

- *Do you feel you can now manage your bronchiectasis with help from your GP if you are sick?*
- *What problems do you see in the future that might make it difficult to manage your bronchiectasis?*
- *Do you know where to get help and advice in the future if you need it?*
- *Do you refer to your written bronchiectasis action plan for ways of managing your condition? (for example, the written airway clearance plan on the back)*
- *Was there enough time to teach you the skills you needed to manage your bronchiectasis? (for example, was enough time spent on teaching you how to clear sputum/phlegm so that you feel confident doing it at home)*

Do you have any other suggestions or anything else you would like to say about your experience of coming to the bronchiectasis clinic?

**Additional results**

The supplementary table S1 outlines the clinic interventions received by the study participants.

The supplementary figure S1 describes the process taken by the research team to define and refine the themes developed from the interview transcripts.

**Table S1 Traits identified and treatments implemented at the outpatient bronchiectasis clinic for study participants, n=10**

| **Trait (n=number of participants)** | **Treatment (n=number of participants)** |
| --- | --- |
| **Pulmonary** |  |
| Mucus hypersecretion (n=10) | Airway clearance therapy +/- nebulised saline (n=10)  Education on hydration- target urine colour (n=10) |
| Dyspnoea (n=5) | Pulmonary rehabilitation, activity pacing/modification (n=4, n=1 declined pulmonary rehabilitation) |
| Cough (n=1) | Treat underlying cause, e.g. mucus hypersecretion (n=1) |
| Infection (n=6) | Airway clearance therapy (n=6)  Education (n=6)  Prompt treatment of exacerbations- education, letter to GP (n=6) |
| **Extrapulmonary** |  |
| Rhinosinusitis (n=3) | Sinus rinse (n=3)  Education regarding nasal steroids, antihistamines, saline sprays (n=3) |
| Urinary incontinence (n=1) | Teach the ‘knack’ (n=1)  Optimise positioning for airway clearance (n=1)  Reduce the use of cough (n=1)  Referral to pelvic health physiotherapist (n=1) |
| Overweight (n=2) | Referral to Dietitian (n=2) |
| Underweight (n=1) | Referral to Dietitian (n=1) |
| Cardiac history (n=2) | Modify exercise prescription (n=2) |
| GORD (n=6) | Modified positioning for airway clearance therapy (n=6)  Referral for assessment- GP (n=1) |
| Other significant medical history (n=1) | Modify interventions as required e.g. exercise (n=1) |
| Pain (n=3) | Modify interventions as required (n=3)  Postural education (n=3)  Exercise (n=2)  Physical activity (n=3) |
| **Environmental/Lifestyle** |  |
| Reduced exercise capacity (n=9) | Referral to pulmonary rehabilitation (n=9, n=1 declined pulmonary rehabilitation)  Home exercise program (n=1) |
| Absence of action plan (n=10) | Develop a Bronchiectasis Action Plan (Lung Foundation Australia) (n=10)  Education- disease specific (n=10) |
| Poor inhalation technique (n=4) | Practise of inhaler technique (n=4)  Use of spacer (n=4) |
| Reduced physical activity (n=5) | Physical activity education (n=5) |
| Sedentarism (n=4) | Break bouts of sedentary time (n=4)  Increase physical activity (n=5) |
| Family and social support (n=3) | Referral to social work (n=3) |

**Note:** At the time of interview completion, participants had not attended the appointments scheduled with health professionals external to the bronchiectasis clinic (e.g. pulmonary rehabilitation, dietitian)

**FINAL THEMES**

**THIRD THEMES**

**SECOND THEMES**

**INITIAL THEMES**

I have a toolbox

The clinic interventions are individualised and highly valuable

Motivation to engage and change

A tool kit to support my health

Multi interventional clinical care is highly valuable

Get it right

Better health, better self

Positive impact of attending the clinic

Psychosocial and physical health benefits from the clinic

Better health, better self

Communication is king

The experience of attending the clinic

I'm in for the long term

Self management is accepted by some, not all

The clinic supported an understanding and acceptance of self- management

Living with bronchiectasis

Persistent challenges of living with bronchiectasis

Some things don't change

**Figure S1 The process of theme development**

**Note:** Codes initially included in the themes ‘Get it right’, ‘Communication is king’, ‘The experience of attending the clinic’ have been incorporated into other themes within the process of iterative theme development.

**References**

Lung Foundation Australia. (2020 Feb). My Bronchiectasis Action Plan. Retrieved June 24, 2022, from <https://lungfoundation.com.au/wp-content/uploads/2020/04/Flyer-Bronchiectasis-Action-Plan-Feb-2020.pdf>.

Sekhon, M., Cartwright, M. and Francis, J. J. (2017). Acceptability of healthcare interventions: an overview of reviews and development of a theoretical framework. BMC Health Services Research, 17(1): 88.
